# Supplementary material for: Microtubule Engagement by the WD40-Containing Tail of Kinesin-4 KIF21B
Source: bioRxiv. 2026 Jul 30:2026.07.29.741364. Preprint. [Version 1] doi: 10.64898/2026.07.29.741364 (PMC13484071; doi:10.64898/2026.07.29.741364)
Supplement: Supplementary file 1 [file NIHPP2026.07.29.741364v1-supplement-1.pdf]

bioRxiv preprint doi: https://doi.org/10.64898/2026.07.29.741364; this version posted July 30, 2026. The copyright holder for this preprint (which was not certified by peer review) is the author/funder, who has granted bioRxiv a license to display the preprint in perpetuity. It is made available under a CC-BY 4.0 International license.

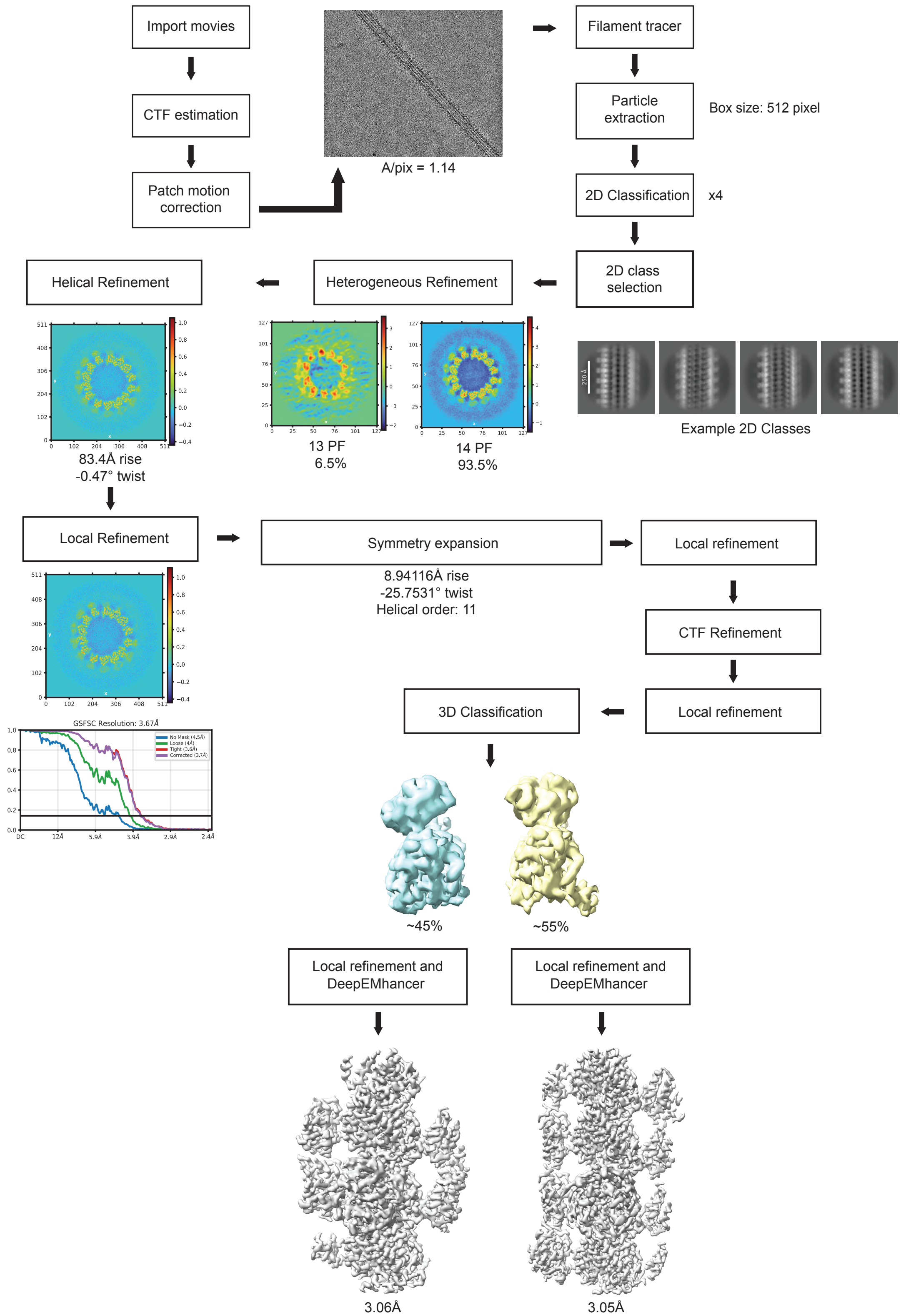

Supplementary Figure 1. | Cryo-EM Processing Pipeline. Overview of cryo-EM processing leading to the final reconstructions of KIF21B-bound MTs.

bioRxiv preprint doi: https://doi.org/10.64898/2026.07.29.741364; this version posted July 30, 2026. The copyright holder for this preprint (which was not certified by peer review) is the author/funder, who has granted bioRxiv a license to display the preprint in perpetuity. It is made available under a CC-BY 4.0 International license.

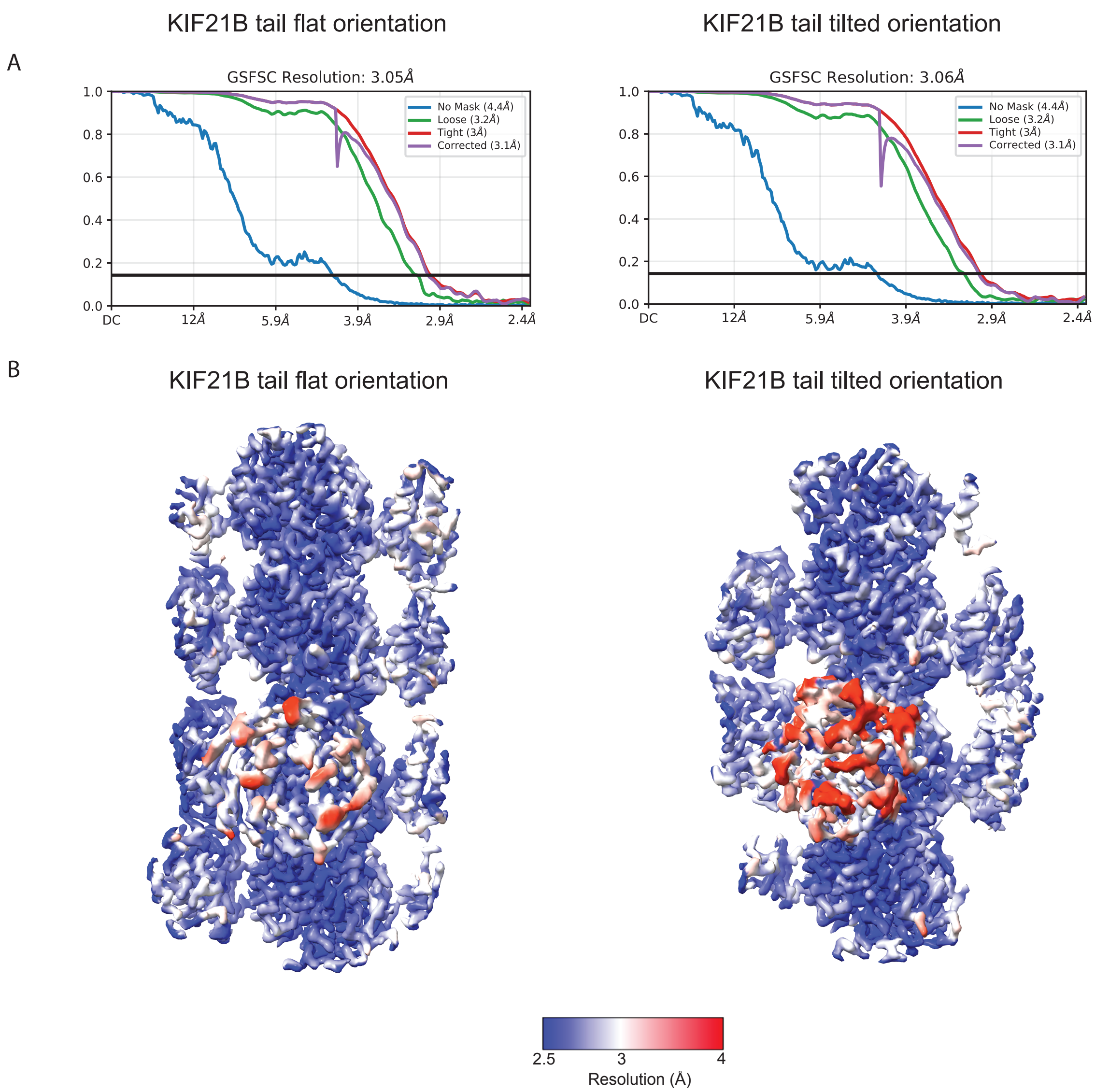

Supplementary Figure 2. | Local resolution mapping and FSCs. (A) FSC plots of the two MT bound KIF21B structures. (B) Local resolution maps of the two MT bound KIF21B structures

bioRxiv preprint doi: https://doi.org/10.64898/2026.07.29.741364; this version posted July 30, 2026. The copyright holder for this preprint (which was not certified by peer review) is the author/funder, who has granted bioRxiv a license to display the preprint in perpetuity. It is made available under a CC-BY 4.0 International license.

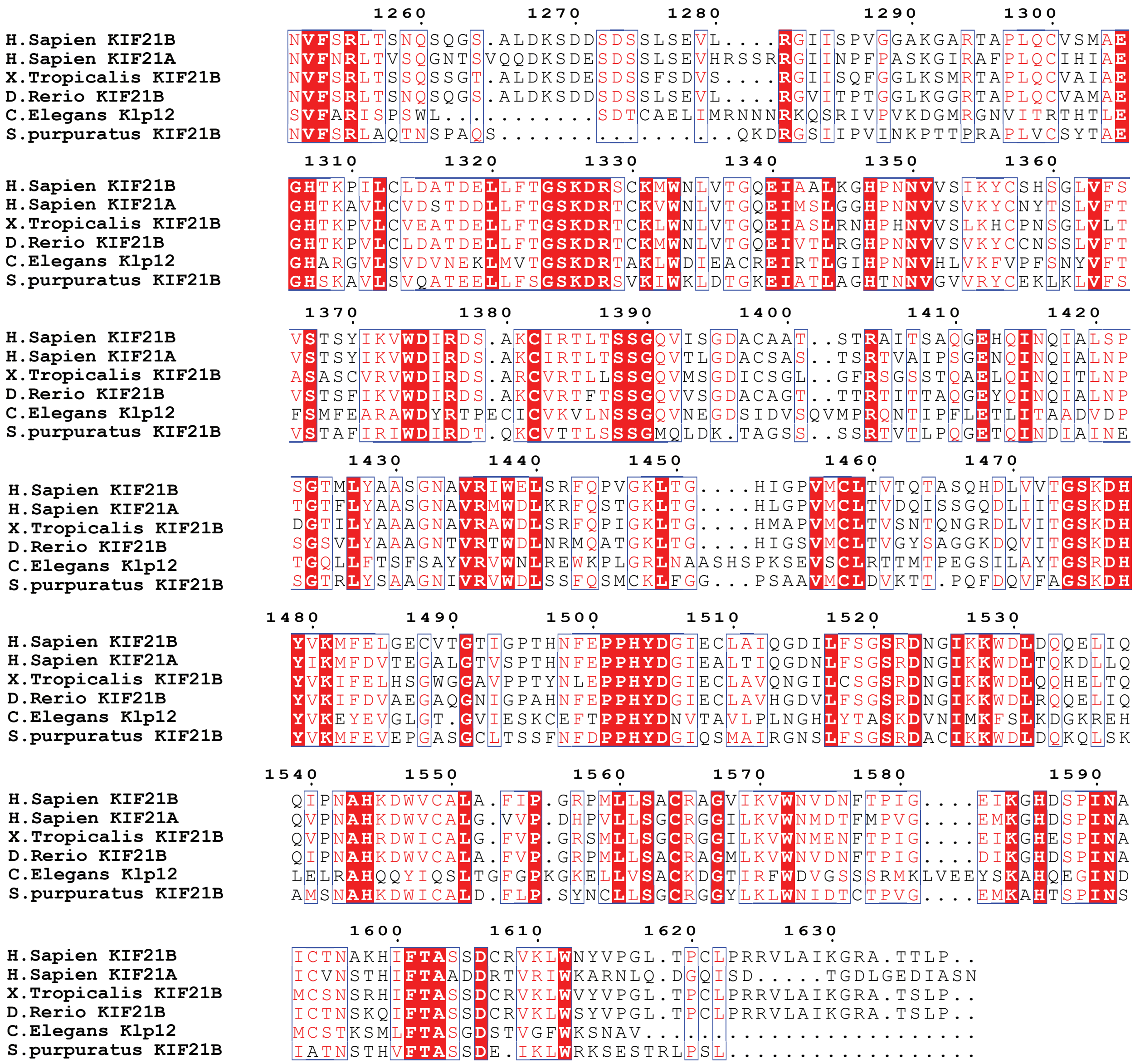

Supplementary Figure 3. | Sequence alignment of the KIF21B tail. Sequence alignment of the KIF21B and KIF21A tail across species. Alignment was displayed using ESPript3 (Robert et al., 2025).
